# Controlling the Organization of Colloidal Sphero-Cylinders Using Confinement in a Minority Phase

**DOI:** 10.3390/gels4010015

**Published:** 2018-02-02

**Authors:** Niek Hijnen, Paul S. Clegg

**Affiliations:** School of Physics & Astronomy, University of Edinburgh, Peter Guthrie Tait Road, Edinburgh EH9 3FD, UK; Niek.Hijnen@akzonobel.com

**Keywords:** colloid, wetting, capillarity

## Abstract

We demonstrate experimentally that a phase-separating host solvent can be used to organize colloidal rods into different cluster and network states. The rods are silica sphero-cylinders which are preferentially wet by the water-rich phase of an oil–water binary liquid system. By beginning with the rods dispersed in the single-fluid phase and then varying the temperature to enter the demixed regime, a precisely chosen volume of water-rich phase can be created. We then show how this can be used to create independent clusters of rods, a percolating network, a network of clusters or a system that undergoes hindered phase separation. These different modes are selected by choosing the relative volumes of the rods and the water-rich phase and by the timing of the temperature change.

## 1. Introduction

Non-spherical colloids move, assemble, and percolate differently compared to standard spherical particles [1,2,3]. Because many natural and synthetic particles, e.g., mineral particles, bacteria, viruses, graphene, carbon nanotubes and fibres are non-spherical, understanding and controlling the new behaviour is valuable [4]. Much has already been achieved by tuning mutual interactions via surface charge or the addition of depletants [5]. Less explored is the approach of organizing non-spherical particles using a phase transition in the host solvent. Low concentrations of rods or platelets can be corralled into a percolating arrangement driven by the phase-separation kinetics. Here, we are considering the host solvent to be a binary mixture of polymers or low molecular weight liquids. The corral is created by the preferred fluid domain or by the interface between the two fluids. Interfacial trapping requires considerable control over the wettability of the colloids [6,7]; by contrast, confining rods or platelets to one of the fluid domains should be more straightforward.

We focus here on colloidal sphero-cylinders as model rod-shaped particles; we are interested in their behaviour as they are forced into a confining volume of solvent. Onsager showed that a population of rods of aspect ratio, *A*, will exhibit a nematic phase for volume fractions, $\Phi_{ons}$, above $\Phi_{ons}A=3.29$ due to the combined effect of the decrease in entropy when the rods align and the excluded volume associated with the relative orientation of the rods. This threshold is valid even down to modest aspect ratios [8,9]. As the volume fraction of rods is further increased, we expect to find [10,11] the maximum for amorphous packing, $\Phi_{a}$, to be described by $\Phi_{a}A=5.1$ and the maximum for ordered packing, $\Phi_{max}$, at

(1) $$\Phi_{max}=\left(\frac{\pi }{\sqrt{27}}+\frac{\pi }{\sqrt{12}}A\right)/\left(\sqrt{\frac{2}{3}}+A\right)\approx \frac{0.6+0.9A}{0.8+A}.$$

At quite low volume fractions, a fine network of ‘sticky’ sphero-cylinders can form and percolate across the sample. A homogeneous network is a random collection of evenly distributed rods, whereas a heterogeneous network is comprised of ramified clusters with a fractal appearance. For volume fractions, $\Phi_{p}$, less than $\Phi_{p}A\approx 0.7$, the network will need to be heterogeneous in order to percolate [12].

Computer simulations by Peng et al. first demonstrated that a percolating arrangement of rod-shaped particles could be formed during a demixing process [13]. The rods were preferentially wet by the minority phase and had highly anisotropic interactions; the resulting networks had enhanced mechanical and electrical properties [14]. Hore and Laradji modeled the behaviour of purely repulsive colloidal rods which also partitioned into a single domain during phase separation [15]. They found that the phase separation process could be dramatically slowed; although arrest in a percolating structure was not observed for the compositions studied on the timescale of the simulations. Experimentally, it has been possible to create a percolating arrangement of nano-rods in a phase-separating polymer mixture. For sufficiently high concentrations of ‘sticky’ nanoparticles, Li et al. found that a continuous percolating domain of rods formed during phase separation [16]. More recently, Xavier and Bose studied the behaviour of multi-walled carbon nanotubes in a phase separating polymer mixture [17]. At the low concentrations employed, the kinetics were strongly modified but the system underwent complete separation.

**Figure 1:**
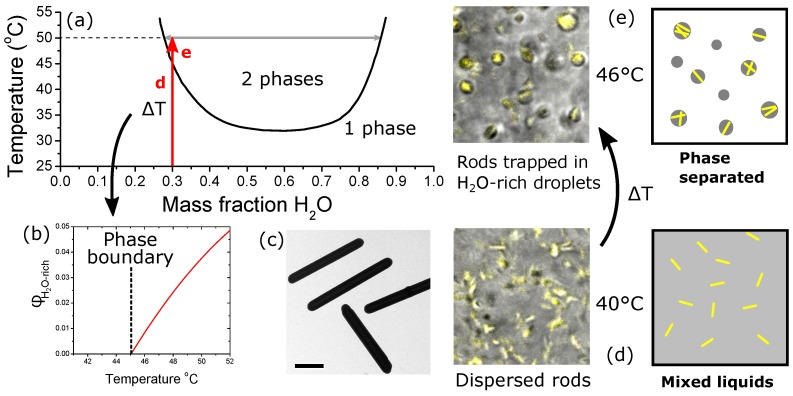
Showing how phase separating water and 1-propoxy-2-propanol can be used to create different phase volumes by controlling the depth of the temperature quench. (**a**) Our partially miscible solvents exhibit a lower critical solution temperature; (**b**) the temperature step into the demixed region can be used to control the volume of the minority phase, here the water-rich phase; (**c**) We use colloids shaped like sphero-cylinders, scale bar 1 μm; (**d**,**e**) The colloids, shown in yellow, are dispersed at low temperature below the binodal line; see red d in (**a**), where the liquids are mixed. On warming the samples, the liquids demix and the colloids are confined within the minority phase; see red e in (**a**).

The purpose of this Communication is to demonstrate that phase separation (see Figure 1a,b) can be used to organize anisotropic particles which preferentially partition into one of the phases. To do this, we carry out experiments using dispersions of colloidal rods (see Figure 1c) in a partially miscible host solvent. We find that a percolating network forms from a small quantity of rods provided that the volume fraction, $\Phi_{v}$, of rods in the water-rich phase exceeds 66%. At significantly lower $\Phi_{v}$, the rods form isolated clusters, following phase separation, that rapidly sediment to the base of the vial. If $\Phi_{v}$ is only slightly below the 66% threshold, then the system becomes exquisitely sensitive to the quench route. Finally, slowly but decisively destabilizing the percolating network leads to a sudden transition from a collapsing network to hindered phase separation. We show that the different outcomes can be precisely controlled.

## 2. Results and Discussion

**Figure 2:**
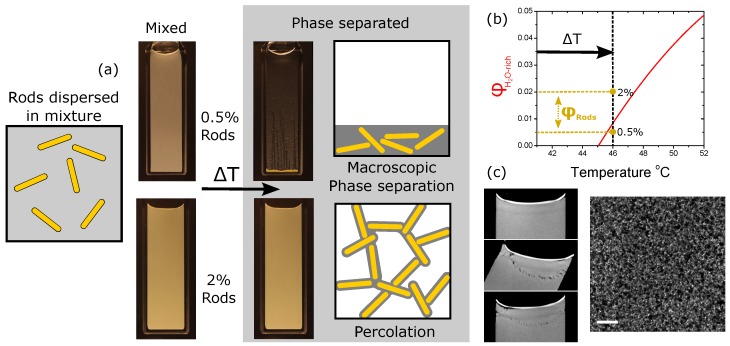
Showing how different concentrations of rods lead to radically different behaviour. (**a**) Images and cartoons of samples with rod volume fractions which are smaller and significantly larger than the volume of the minority phase; (**b**) The variation of the minority phase volume as a function of temperature compared to the volume of rods in the two cases of interest; (**c**) Image of a sample with 2% rods as it is tipped. There are indications that flow is resisted. The confocal micrograph shows the internal network formed by the rods, scale bar 50 μm.

First we describe experiments using the lowest volume fractions of rods, Figure 1c. The sample is quenched from room temperature to 46 ${}^{\circ }$C by submerging it in a thermostated water bath. The choice of liquids and particles is outlined in Materials and Methods, below. Inside the vial [18], the sample changes temperature at a rate of 1 ${}^{\circ }$C/s. This shallow quench leads to liquid–liquid phase separation and the formation of 1 vol. % of water-rich phase (see Figure 2a). If 0.1 vol. % of rods are added to the sample at the beginning of the experiment, then the liquids separate by nucleation and growth and the rods are observed in the droplets after the transition (see Figure 1d,e). The silica surfaces are coated with silanol groups and a layer of physically bound water; it is no surprise that they are hydrophilic [19]. The water-rich phase is the more dense and so the rod-filled droplets eventually collect at the base of the vial. Qualitatively, the same thing is observed when 0.5 vol. % of rods are used. Immediately following the quench, the sample appears macroscopically homogeneous; however, within 2 h, all of the clusters of particles have sedimented to the base of the vial. This sample composition gives a volume fraction of $\Phi_{v}\approx 33$% in the water-rich phase, Figure 2b. It is perhaps surprising that we observe little that is special due to the shape of the particles in this regime given that the 0.5 vol. % sample should be approaching the isotropic to nematic transition during the phase separation process ($\Phi_{ons}=34$%).

Maintaining the same quench depth (i.e., to 46 ${}^{\circ }$C) but raising the volume fraction of rods to 2 vol. % yields entirely different behaviour. Superficially, following the quench, the sample again appears macroscopically homogeneous. Now, however, on tipping it becomes immediately obvious that the sample has a solid-like character, Figure 2a,c. Evidently, the combination of phase separation and the 2 vol. % of colloidal particles ($\Phi_{v}\approx 67$%) are conveying a yield stress to the sample. Microscopic observations, Figure 2c, reveal a fine network of colloidal rods which percolate across the sample. There are no obvious droplets of water-rich phase; instead, the solvent appears to tightly envelop the particles. This is very similar to the case of gels held together by capillary bridges [20,21]. This phenomena has also been studied using rods and fully immiscible liquids [22]; no network formation was found in that case. With our rods and partially miscible fluids, we find that the system’s attempt to lower the liquid–liquid interfacial area leads to significant effective attractions between the rods. It is well known that such attractive interactions greatly enhance the likelihood of percolation [23].

Networks formed from colloidal rods and nanotubes have been categorized as homogeneous or heterogeneous depending on whether the components are evenly distributed [12,23]. The micrographs show a ‘spikey’ network with some strands much longer than the individual rods, Figure 2c; qualitatively, this is consistent with the idea of a heterogeneous network. Quantitatively, we expect that our rods will only be able to percolate at volume fractions below $\Phi_{p}\approx 7$% via the formation of a heterogeneous network [12]. Because the combined volume of the water-rich phase and particles make up 3 vol. % of the sample, this is consistent. Within the water-rich phase, the volume fraction of rods is larger than that expected for the highest density amorphous packing, $\Phi_{a}\approx 50$%. Hence, we anticipate that the liquid domain has pulled the particles together in a disordered arrangement of a liquid envelope combined with capillary bridges, Figure 2a,b. The latter are responsible for giving the network its strength.

**Figure 3:**
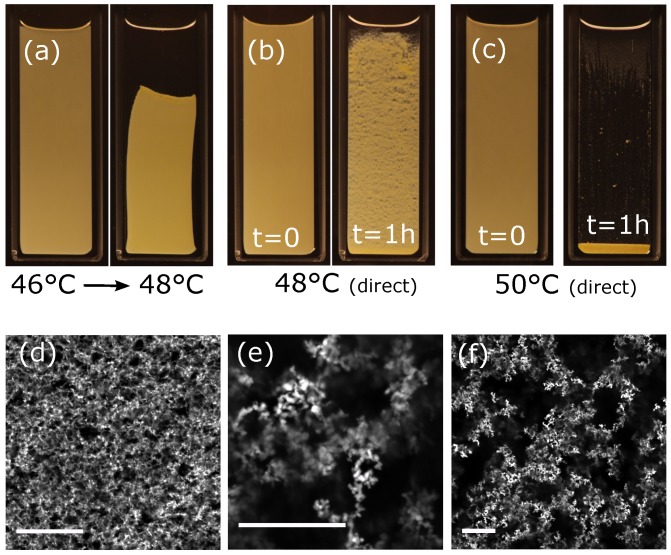
(**a**) Sample of 2 vol. % rods (left panel) first immersed in a water bath at 46 ${}^{\circ }$C, and subsequently (right panel) slowly heated in the water bath to 48 ${}^{\circ }$C; (**b**) Sample of 2 vol. % rods immersed in a water bath at, 48 ${}^{\circ }$C, (left panel) immediately, and (right panel) one hour after immersion; (**c**) Sample of 2 vol. % rods immersed in a water bath at 50 ${}^{\circ }$C, (left panel) immediately, and (right panel) one hour after immersion; (**d**–**f**) Confocal laser scanning microscopy images taken at 48 ${}^{\circ }$C of samples shown in (**a**) confocal image (**d**) and (**b**) confocal images (**e**,**f**). Note changes in the 100 μm scale bars.

We now increase the depth of the quench, finishing at a temperature of 48 ${}^{\circ }$C, while keeping the volume fraction of rods at 2 vol. % and we show how the behaviour depends on the timing of the temperature changes. Increasing the final temperature increases the volume of the water-rich phase, here to 2 vol. %. Macroscopically, the sample is initially homogeneous; however, within an hour, it has sedimented slightly and it is clearly non-uniform, Figure 3b. Microscopically, the network now looks quite different to the one formed by the same quantity of particles in a quench to 46 ${}^{\circ }$C. The strands of the network are thicker and there are significant gaps between different sections of the network, Figure 3e,f. For comparison, we now take a sample of identical composition to 48 ${}^{\circ }$C by a different route. We first quench the sample to 46 ${}^{\circ }$C to form a stable network and then it is warmed in the bath to 48 ${}^{\circ }$C. The stable network, Figure 3a left panel, macroscopically sags and collapses noticeably under its own weight, Figure 3a right panel. The changes on the microscopic scale are much less significant compared to the alternative heating route: some bright droplets are evident and some narrow network threads may have broken, Figure 3d. Evidently, reducing the volume fraction of rods within the water-rich phase to $\Phi_{v}\approx 50$% has prevented the composite from forming a stable network without first sedimenting.

The relationship between sedimentation and aggregation has been considered by Allain et al. [24]. For a sufficiently high concentration of attractive particles, they demonstrate that a percolating network will form without settling due to gravity, i.e., aggregation will beat sedimentation. This is reminiscent of the situation for 2 vol. % of rods quenched to 46 ${}^{\circ }$C. For a lower concentration of particles, the relative importance of sedimentation and aggregation is more finely balanced. They show that, initially, clusters of attractive particles will form that will not percolate; instead, the clusters will sediment and eventually form a percolating network of clusters which will not occupy the sample to the top [24]. This seems to accurately capture the behaviour of 2 vol. % of rods quenched directly to 48 ${}^{\circ }$C. The significantly different appearance of the network is consistent with pre-formed clusters of rods having subsequently connected to span the sample. The boundary between network formation and cluster formation has been probed in detail for spherical particles and fully immiscible liquids by Heidlebaugh et al. [25]. In our experiments, these two scenarios have been observed for identical concentrations of particles but different volumes of the water-rich phase. The very different behaviour of these two systems is driven by the fact that a quench to 48 ${}^{\circ }$C creates a larger volume water-rich phase which allows the rods to reorganize. The rods first make contact and then align in bundles and it is these bundles that are tightly enough enveloped in the water-rich phase to be ‘sticky’. The bundles cluster, sediment and eventually form the network of clusters. This creates an effective change in the particle concentration. The change in thickness of the network strands is visible in Figure 3e,f.

**Figure 4:**
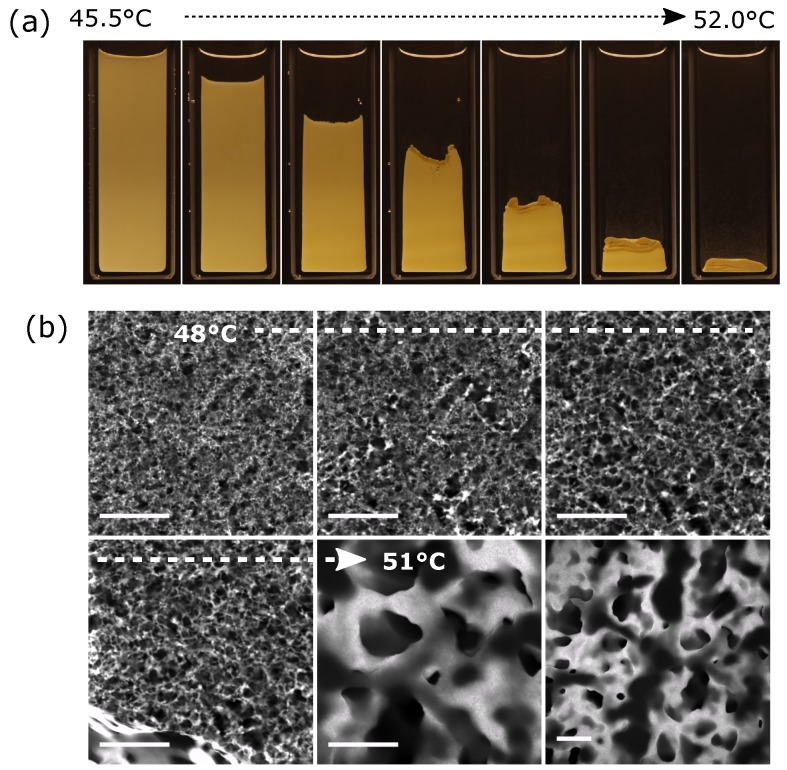
(**a**) A sample (2 vol. % rods) quenched to 45.5 ${}^{\circ }$C, $\Phi_{v}\approx 80$%, was then slowly heated to 52 ${}^{\circ }$C, $\Phi_{v}\approx 30$%; (**b**) A time series of confocal micrographs showing (panel 1) the structure of a similar sample at 48 ${}^{\circ }$C, which is subsequently heated (panels 2–5) to 51 ${}^{\circ }$C. The bottom right panel is a lower magnification image of the structure in panel 5. All scale bars 100 μm.

Finally, we demonstrate that a sharp transition occurs when there is a very significant rise in the water-rich phase volume, Figure 4. Here, an initial stable network has been created by quenching 2 vol. % rods into the demixed regime. This network is then steadily destabilized by gentle warming. In Figure 4a, the self-supporting network is seen to steadily collapse as the volume of the water-rich phase is increased. Ultimately, only a water-rich phase, densely packed with rods, remains at the base of the vial. Microscopically, we begin to see small local changes to the network as it is warmed, Figure 4b panels 2–3. Small bright droplets become increasingly prevalent and the mesh size of the network increases noticeably. Approaching $\Phi_{v}\approx 30$% there is a sudden change where the network is replaced by a large fluid domain. From this point onwards, the two liquid/particle system now resembles a conventional phase separation, Figure 4b panel 6. As predicted using computer simulations [15], the coarsening of the domain pattern is extremely slow due to the presence of the rods.

The sudden change from network to rod-filled liquid domain, Figure 4, is a consequence of the change in the rod–rod interactions. For networks formed from $\Phi_{v}>66$%, there are no rearrangements; the structure is self-supporting and robust. It appears that the water-rich phase envelope around the rods is responsible for strong, short-range attractions which are akin to the effect of a primary minimum in the interaction potential. We believe that the junctions between rods have some resemblance to capillary bridges [20,21]; although, our system is quite different because the rods are fully coated with the water-rich phase. Once the water-rich phase volume increases, the apparent strong attractions no longer control the system and a liquid domain filled with rods then emerges. The flow and accompanying slow coarsening only begin once the dominance of capillarity has ended.

## 3. Materials and Methods

The binary fluid system used here is a mixture of water and 1-propoxy-2-propanol (PGPE), Figure 1a [26]. The rods are prepared by a multi-step route [27] that yields hollow cylinders of length 3.5 μm, aspect ratio A=9.7 with $\sigma_{A}=16$%. To begin with, the rods are dispersed in the single-fluid phase at room temperature, typically with a volume fraction in the range 0.5–2%. Away from the phase boundary, the rods remain well dispersed demonstrating that they repel one another in the single-fluid phase. Over a period of 24 h, the rods sediment to the base of the vial; our experiments are usually carried out within 2 h unless otherwise mentioned above and hence are not greatly affected. It is important to note that the behaviour is very different close to the phase boundary. We have previously studied this in some detail [28] and we avoid this region here. In the experiments described above, all samples contain 30 wt % water in PGPE; we study the behaviour of the rods after quenching to different depths in the two-fluid phase via a change in temperature. Using the Lever rule, the change in temperature can be converted into a volume fraction of water-rich phase (see Figure 1b). This is the phase into which the rods always partition.

## 4. Conclusions

To conclude, we have shown that a well-controlled volume of the water-rich phase, created via demixing, can direct the organization of colloidal rods to form different structures and domains. While the water-rich phase in our experiments never occupies more than a few percent of the total sample volume, the concentration of rods within this phase can become very high. This creates effective attractive interactions between the rods leading to the formation of clusters, networks, networks of clusters and phase separating domains as the concentration is varied. Hence we have demonstrated that phase separation can be used to organize anisotropic particles which preferentially partition into one of the domains. Being able to trigger clustering, network formation, etc., via a sudden change in temperature and the relative insensitivity to the precise wetting properties of the particles are the strengths of this approach.
